# Employee attitudes toward suicide prevention and Counseling on Access to Lethal Means: initial findings from an academic medical center implementing the Zero Suicide framework

**DOI:** 10.3389/fpubh.2023.1268300

**Published:** 2023-11-03

**Authors:** Rachael A. Jasperson, Emily Sullivan, Evan V. Goldstein

**Affiliations:** ^1^Zero Suicide Program, University of Utah Health, Salt Lake City, UT, United States; ^2^Hunstman Mental Health Institute, University of Utah, Salt Lake City, UT, United States; ^3^Department of Population Health Sciences, Spencer Fox Eccles School of Medicine, University of Utah, Salt Lake City, UT, United States

**Keywords:** suicide, self-injurious behavior, health personnel, attitude of health personnel, firearms

## Abstract

**Introduction:**

Zero Suicide is a strategic framework designed to transform a healthcare system’s suicide prevention activities. In 2020, University of Utah Health launched a Zero Suicide program and Counseling on Access to Lethal Means (CALM) training for its employees. In 2022, the healthcare system surveyed its workforce’s attitudes toward suicide prevention and CALM. We sought to evaluate employees’ attitudes and behaviors toward suicide prevention and CALM training following the launch of the Zero Suicide program.

**Methods:**

A Zero Suicide Workforce Survey was administered online through REDCap to all University of Utah Health employees. The analytic sample included 3,345 respondents. We used two-portion z-tests to compare the proportions of respondents who (1) completed CALM training and (2) did not yet complete the CALM training because they felt it was irrelevant to their position by different employee characteristics.

**Results:**

More than half of the respondents in the analytic sample were directly interacting with patients who may be at risk for suicide (57.6%). About 8.4% of the respondents had completed CALM training. Among those who had not yet completed CALM training, 9.5% indicated they did not think CALM was relevant to their job. Respondents knowledgeable about warning signs for suicide and respondents who were confident in their ability to respond when suspecting elevated suicide risk were significantly more likely to complete CALM training.

**Discussion:**

This evaluation provides important insights from the workforce of a large academic medical center implementing a Zero Suicide program, including insights on opportunities for improving program implementation and evaluation.

## Introduction

In 2021, suicide was the 11th leading cause of death in the US and the second leading cause of death for individuals ages 10–40 (1). More than 48,000 people died by suicide, more than 12 million seriously considered suicide, and 1.7 million people attempted suicide in the US in 2021. From 2000 to 2018, there was a 30% increase in suicide rates, despite numerous suicide prevention efforts occurring in healthcare and community settings. After two consecutive years of decreases in suicide deaths (47,511 in 2019 and 45,979 in 2020), the most recently available data from 2021 indicated an increase in suicide deaths nearly returning to the recent peak in 2018 (48,344) with an age-adjusted rate of 14.1 suicide deaths per 100,000 people (versus 14.2 per 100,000 people in 2018). Within the US, states located in the Mountain Census Division tend to experience the highest suicide rates (2, 3), including Utah, which had the ninth-highest age-adjusted suicide rate among all states from 2018 to 2021 (1).

The scholarly literature has long discussed suicide risk factors, such as having a prior suicide attempt, mental health condition, and exposure to prior violence (4, 5). However, suicidal crises are often episodic and brief, and because suicidal crises can escalate rapidly, they are difficult to predict. Most individuals who attempt suicide make the decision to act on that decision in under 60 min, with 48% reporting acting within 10 min (6). What is clear is that the means a person uses during an attempt can have a drastic impact on whether the person survives the attempt. Previous studies have shown that restricting access to lethal means during a time of crisis can positively impact suicide rates (7). This has been shown to be effective in areas such as bridge barriers, detoxification of domestic gas, pesticides, medication packaging, and others (8). To that end, firearms are particularly problematic for suicide prevention. Firearm availability exacerbates suicide risk (9–12), and firearms are the most common (13) and lethal (14) suicide means among US. Many people who attempt suicide ultimately survive (15); however, survival is typically less likely for those who use firearms, given the 80–90% case-fatality rate of firearm attempts (16, 17). Moreover, recent studies on firearms relative to other suicide methods have found that substance use, mental health diagnoses, and prior suicide attempts may be less likely among suicide decedents who die by firearm relative to other methods (18–20).

In 2010, the National Action Alliance for Suicide Prevention (The Action Alliance), a public-private partnership for suicide prevention, was established. The goal of The Action Alliance was to advance the National Strategy for Suicide Prevention by supporting suicide prevention efforts focused on transforming health systems, transforming communities, and changing the conversation about suicide (21). The Action Alliance identified the integration of suicide prevention strategies into healthcare organizations as a priority. The primary objective was the promotion and adoption of “zero suicide” as an aspirational organizational goal (21). As a result, the Clinical Care and Intervention Task Force was created and the Zero Suicide Initiative was born (22).

Zero Suicide is a strategic framework designed to transform a healthcare system’s suicide care (23, 24). It is a systematic approach to quality improvement with the aspirational goal of zero suicide deaths for patients under the organization’s care. The framework provides guidance and resources to support the multilevel implementation and execution of suicide prevention best practices. Zero Suicide operationalizes seven core components that are necessary for transforming a system’s suicide care: *Lead, Train, Identify, Engage, Treat*, *Transition* and *Improve* (24, 25). One of the evidence-based interventions specified in the *Engage* component of the Zero Suicide framework is lethal means reduction in the form of Counseling on Access to Lethal Means (CALM). CALM is an approach to having conversations with individuals at risk for suicide about restricting their access to lethal means – such as firearms and medications – during a suicidal crisis. The focus of CALM is ensuring that dangerous and lethal means are less available should a suicidal crisis occur, as well as decreasing the chances of an attempt and increasing the likelihood of survival should the attempt occur.

In 2020, University of Utah Health – the only academic medical center in Utah – launched a Zero Suicide program. With an emphasis on building a system-wide culture dedicated to dramatically reducing suicides, efforts were put towards developing a competent, confident, and caring workforce equipped to identify, engage, and care for individuals at risk for suicide. These efforts have included employee training in primary areas: Basic suicide prevention education, safety plan development, and CALM. In collaboration with Intermountain Healthcare, University of Utah Health added a one-hour CALM module to its internal employee education system (LMS). In addition, a standard 1-h virtual training was offered monthly through 2021, and then was moved to quarterly. CALM training was completed by all social workers and the LMS module and virtual training were assigned to all new hire social workers. CALM training has also been incorporated into the University of Utah Health medical student education and psychiatric residency program. Additionally, CALM training has been conducted with institution leadership and Emergency Department and primary care providers. By early 2022, prior to the current study’s survey being conducted, 397 people completed the LMS CALM training and 553 attended either virtual or in-person training. Mandating educational requirements in a large healthcare system is often approached with a great deal of organizational scrutiny. Therefore, no institutional policies were implemented mandating suicide prevention or CALM training for staff. Rather, the approach to date has been to implement clinical best practice guidelines and to target integration of CALM into already existing educational opportunities.

In 2022, University of Utah Health administered the first bi-annual survey of its workforce’s attitudes toward suicide prevention and CALM training within the context of the health system’s burgeoning Zero Suicide program. We sought to evaluate employees’ attitudes and behaviors toward suicide prevention and CALM training following the launch of the program. This evaluation of our initial bi-annual survey will help inform subsequent workforce surveys and implementation efforts and yield important insights from an academic medical center implementing a Zero Suicide program.

## Method

### Data and sample

University of Utah Health used a modified version of the Zero Suicide Institute’s workforce survey to measure and establish an understanding of its workforce’s suicide-prevention-related attitudes, knowledge, and culture. It is important to note that system resource utilization was complicated by the onset of the COVID-19 pandemic. The initial workforce survey data were gathered 2 years following the launch of the Zero Suicide program at University of Utah Health. Data were gathered through the initial survey from March 29, 2022, to April 20, 2022. The analyzes presented in this paper were then completed in 2023. This study involving human subjects was reviewed and received an exemption determination from the University of Utah Institutional Review Board for Federal Exemption Categories 2 and 4 defined in 45 CFR 46.101(b) (IRB_00142911).

The 2022 Zero Suicide Workforce Survey was administered online through REDCap – a secure web-based application – to all individuals employed by University of Utah Health and the School of Medicine. Completion of the survey was voluntary and anonymous. On average, completing the survey took less than 5 min. Out of approximately 22,500 eligible employees, 2,919 fully completed the survey (13.0% of eligible employees). An additional 426 responses were imputed using multiple imputation by chained equations to replace missing data using variables containing non-missing observations for respondents who partially completed the survey. The final analytic sample included 3,345 respondents (representing 14.9% of eligible employees).

### Measures

The survey included 10 questions, with two questions containing multiple sections, to assess the respondents’ understanding of the Zero Suicide initiative and their attitudes and behaviors related to suicide prevention and CALM. Two outcomes were of primary interest for this evaluation. The first outcome was a binary measure equal to 1 if a respondent indicated that they completed CALM training online or in person and 0 if not. The second outcome was a binary measure equal to 1 if a respondent did not complete the CALM training because they did not believe CALM was relevant for their job and 0 if not for this reason.

We examined differences in the outcomes by four other factors. First, whether or not a respondent was involved in direct patient care. Second, whether or not a respondent interacted with patients who may be at risk for suicide in person or from a distance in their day-to-day duties (e.g., answering phones, scheduling appointments, conducting check-ins, and providing caregiving and/or clinical services). Third, whether or not a respondent believed that they are knowledgeable about warning signs for suicide. Fourth, whether or not a respondent was confident in their ability to respond when they suspect an individual may be at elevated risk for suicide. Each variable equaled 1 if a respondent answered “Yes” or 0 if a respondent answered “No.”

### Statistical analysis

We summarized the distribution of each variable of interest for all respondents in the analytic sample. We used two-portion z-tests to compare the proportions of respondents who (1) completed the CALM training and (2) chose not to complete the CALM training because they felt it was not relevant to their position by the four binary factors described above. We estimated logistic regression models to further examine the relationships between these factors and our two outcomes of interest, presenting the model coefficients as odds ratios for ease of interpretation. An *a priori* significance level of 0.05 was established. All analyzes were conducted using Stata MP version 17.1 (College Station, TX).

## Results

More than half of the respondents in the analytic sample were in primary professional roles involving direct patient care (56.5%) and directly interacting with patients who may be at risk for suicide (57.6%; Table 1). About 66.9% of the respondents reported that they were knowledgeable about warning signs for suicide, though only 55.3% of the respondents were confident in their ability to respond when suspecting elevated suicide risk. About 8.4% of the respondents completed CALM training. Among those who had not yet completed CALM training, 3.9% indicated that they planned to complete CALM training; however, 9.5% indicated they thought CALM was irrelevant to their job.

**Table 1:** Describing the respondents’ attitudes and behaviors related to suicide prevention and CALM (*n* = 3,345).

	Count	Percent
*Respondent’s primary professional role involves direct patient care*		
No	1,456	43.5%
Yes	1,889	56.5%
*Respondent directly interacts with patients who may be at risk for suicide*		
No	1,417	42.4%
Yes	1,928	57.6%
*Respondent is knowledgeable about warning signs of suicide*		
No	1,107	33.1%
Yes	2,238	66.9%
*Respondent is confident in their ability to respond when suspecting elevated suicide risk*		
No	1,494	44.7%
Yes	1,851	55.3%
*Respondent completed the CALM training*		
No	3,064	91.6%
Yes	281	8.4%
*Respondent plans to complete the CALM training (among those who did not yet complete the CALM training)*		
No	2,944	96.1%
Yes	120	3.9%
*Respondent believes CALM is not relevant for their job (among those who did not yet complete the CALM training)*		
No	2,772	90.5%
Yes	292	9.5%

Table 2 describes differences in the proportions of respondents in our analytic sample who completed CALM training by the four binary factors described above. For ease of interpretation, the proportions are expressed as percentages. More than one in ten respondents (11.8%) whose primary professional role involved direct patient care completed CALM training, compared to 3.9% of respondents not involved in direct patient care (*p* < 0.001). Around 11.4% of respondents who reported being knowledgeable about warning signs for suicide completed CALM training, compared to 2.4% of respondents who were not knowledgeable about suicide warning signs (*p* < 0.001). About 12.5% of respondents who reported being confident in their ability to respond when suspecting elevated suicide risk completed CALM training, compared to 3.4% of respondents who were not confident in their ability to do so (*p* < 0.001).

**Table 2:** Describing the respondents who completed the CALM training (*n* = 3,345).

	Percent of respondents who completed CALM training	*p* value
*Respondent’s primary professional role involves direct patient care*		
No	3.9%	<0.001
Yes	11.8%	
*Respondent directly interacts with patients who may be at risk for suicide*		
No	4.0%	<0.001
Yes	11.6%	
*Respondent is knowledgeable about warning signs of suicide*		
No	2.4%	<0.001
Yes	11.4%	
*Respondent is confident in ability to respond when suspecting elevated suicide risk*		
No	3.4%	<0.001
Yes	12.5%	

All 3,345 in the analytic sample were included in this analysis.

As shown in Table 1, most respondents in the analytic sample (91.6%) did not yet complete CALM training. Among the respondents who did not complete the training, and of those respondents involved in direct patient care, 4.9% believed CALM was irrelevant to their job (Table 3). Comparatively, 15.0% of the respondents who did not complete the training and were not involved in direct patient care believed CALM was irrelevant to their job (*p* < 0.001). In addition, about 8.0% of the respondents who did not complete the training and who were confident in their ability to respond when suspecting elevated suicide risk believed CALM was irrelevant to their job, compared to 11.3% of respondents who did not complete the training and were lacking confidence in their ability to respond (*p* = 0.002). Finally, around 9.0% of the respondents who did not take the training but who were knowledgeable about warning signs for suicide believed CALM was irrelevant to their job, which did not differ statistically from the percentage of respondents who did not take the training and were not knowledgeable about suicide warning signs (*p* = 0.154).

**Table 3:** Describing the respondents who did not complete the CALM training because they believe CALM is not relevant to their job (*n* = 3,064).

	Percent of respondents who did not yet complete CALM training because they believe CALM is not relevant to their job	*p* value
*Respondent’s primary professional role involves direct patient care*		
No	15.0%	<0.001
Yes	4.9%	
*Respondent directly interacts with patients who may be at risk for suicide*		
No	17.3%	<0.001
Yes	3.3%	
*Respondent is knowledgeable about warning signs of suicide*		
No	10.6%	0.154
Yes	9.0%	
*Respondent is confident in ability to respond when suspecting elevated suicide risk*		
No	11.3%	0.002
Yes	8.0%	

All 3,064 respondents who did not yet complete the CALM training were included for this analysis.

**Figure 1:**
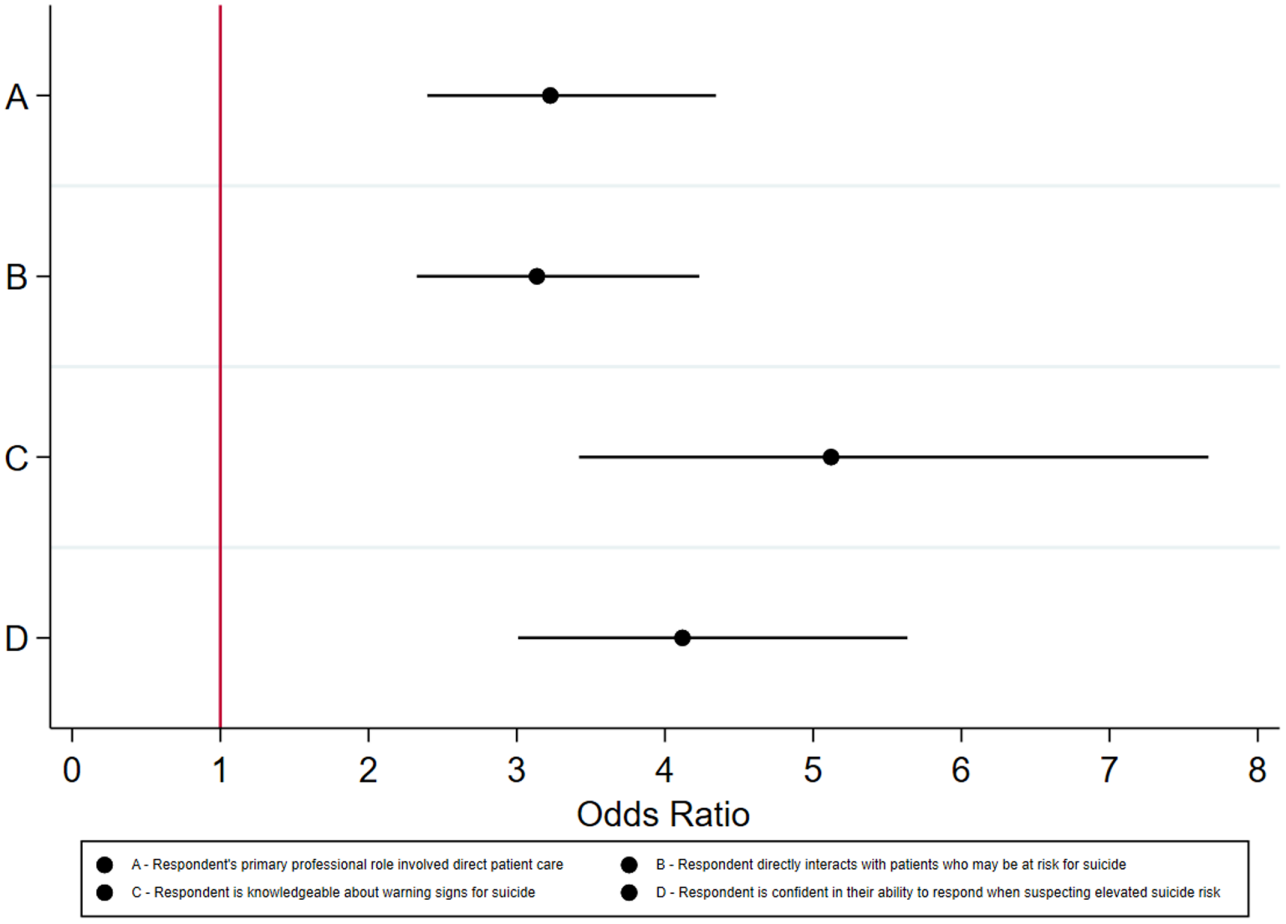
Odds ratios that respondents completed CALM training by different employee characteristics (*n* = 3,345).

Figure 1 describes the unadjusted odds that respondents with varying employee characteristics and knowledge or confidence about suicide prevention completed CALM training. On average, compared to respondents who were lacking confidence in their ability to respond when suspecting elevated suicide risk, respondents with such confidence had 4.12 greater odds of completing CALM training (95% CI: 3.01, 5.64; *p* < 0.001). Respondents who directly interact with patients who may be at risk for suicide had 3.13 greater odds of completing CALM training than respondents who do not directly interact with patients who may be at risk for suicide (95% CI: 2.33, 4.23; *p* < 0.001). Respondents involved in direct patient care had 3.23 greater odds of completing CALM training than those who were not directly involved in patient care (95% CI: 2.40, 4.34; *p* < 0.001). Respondents who reported being knowledgeable about warning signs for suicide had 5.12 greater odds completing CALM training than respondents without such knowledge (95% CI: 3.42, 7.67; *p* < 0.001).

Supplementary Figure S1 shows that respondents involved in direct patient care had 71% lower odds of not completing CALM training because they believed CALM was irrelevant to their job compared to respondents who were not involved in direct patient care (OR = 0.29; 95% CI: 0.22, 0.38; *p* < 0.001). Respondents who did not take the training but who were confident in their ability to respond when suspecting elevated suicide risk also had significantly lower odds of believing CALM was irrelevant to their job compared to respondents who lacked the confidence to respond when suspecting elevated suicide risk (OR = 0.68; 95% CI: 0.53, 0.87; *p* < 0.001). There was no difference between knowledgeable respondents and those who were not knowledgeable about warning signs for suicide in their likelihood of completing the CALM training due to beliefs that CALM was irrelevant to their job (OR = 0.84; 95% CI: 0.65, 1.07; *p* = 0.154).

## Discussion

Despite the initial bi-annual survey’s low response rate, this evaluation provides important insights into attitudes and behaviors toward suicide prevention and CALM training from the workforce of a large academic medical center implementing a Zero Suicide program, as well as insights on opportunities for evaluating the implementation of a Zero Suicide program. About two-thirds (66.9%) of the respondents in our analytic sample were knowledgeable about warning signs of suicide, and over half (55.3%) of the respondents were confident in their ability to respond when suspecting elevated suicide risk. Given the Zero Suicide framework’s emphasis on lethal means reduction, CALM is an integral component of the University of Utah Health’s Zero Suicide program. This analysis suggested that University of Utah Health employees who were knowledgeable about suicide warning signs were significantly more likely to have completed CALM training compared to those who were not knowledgeable about suicide warning signs. Moreover, respondents in our analytic sample who were engaged in direct patient care were over 3.0 times more likely to have completed CALM training compared to respondents not involved in direct patient care. Employees with the most frequent contact with individuals at risk for suicide were the primary target population for CALM training at University of Utah Health. These employees are predominantly behavioral health personnel and emergency department and primary care providers. Social workers comprised the largest segment of University of Utah Health clinicians completing CALM training. The heightened awareness of suicide warning signs could likely be attributed to other suicide awareness or prevention training that targeted these groups and not necessarily a result of having engaged in the actual CALM training. In addition to CALM training, the University of Utah Health’s general suicide prevention training reviews suicide warning signs and risk factors and generally addresses how to respond when suspecting a patient is experiencing elevated suicide risk.

Our results underscore several important challenges for academic medical centers attempting to implement a Zero Suicide program with CALM training. First, over 9-in-10 respondents in our analytic sample did not yet complete CALM training at the time the survey was administered. Given that there are no organizational policies in place mandating CALM training, finding ways to engage teams across the system has proven to be a challenge. Second, there was no difference between respondents who were knowledgeable about the warning signs for suicide and those who were not knowledgeable about suicide warning signs in believing CALM training was irrelevant to their job. These findings are concerning for several reasons. Previous studies suggest that lethal means assessment and counseling can help reduce firearm suicide attempts and deaths (7, 26, 27). CALM training has specifically been shown to be an effective strategy for promoting lethal means counseling, increasing providers’ comfort and confidence in discussing access to lethal means with patients, and increasing providers’ knowledge about firearm access as a risk factor for suicide (28, 29). Thus, higher rates of CALM training and practice may help clinicians save lives, especially in a state like Utah, which experienced the eighth-highest age-adjusted firearm suicide rate in the U.S. from 2010 through 2020 (13).

CALM training is also an important patient safety and quality of care issue, which is the responsibility of the system, and which requires an organizational culture within which process gaps and failures can be openly examined for quality improvement opportunities (30). Historically, suicide has been seen as a mental health issue, and suicide prevention efforts have largely been the responsibility of behavioral health practitioners. However, we now know that nearly all people who die by suicide have had contact with a healthcare provider in the year prior to their death and that primary care and medical specialty visits without mental health diagnoses were most common in the month prior to death (31). What this means is that many individuals who died by suicide are being seen by medical professionals without behavioral health specialty training. If suicide were treated like other patient safety and quality concerns, and if more clinicians could acquire CALM training and conduct CALM, could healthcare systems prevent more suicide deaths for patients under their care?

To that end, our findings signal several opportunities. The majority of respondents in our analytic sample who did not complete CALM training (90.5%) believed that CALM was relevant to their job. As prior implementation science research has suggested that it can take years to translate evidence into clinical practice (32), it is reasonable to expect that additional time and implementation efforts will result in many more University of Utah Health employees completing CALM training in the future. Notably, the survey did not explain CALM in detail to the respondents. A lack of understanding may be a contributing factor to those who responded that CALM was not relevant to their jobs. Systematic outreach to University of Utah Health employees and the provision of informational materials about CALM prior to CALM training will likely be critical for improving employees’ understanding of the importance of CALM, especially for employees who do not have previous exposure to other suicide prevention training. Concurrently, additional or alternative workforce survey questions could be developed to allow employees to provide detailed input on perceived barriers to implementing CALM training. Furthermore, it may be useful to augment the bi-annual workforce survey with in-depth employee interviews or focus groups to better diagnose the root causes of employee reluctance or barriers to completing CALM training, whether or not employees understand what CALM is. For example, the Consolidated Framework for Implementation Research (CFIR) has been used in prior studies to collect clinicians’ insights on adopting suicide interventions into practice (33), eliciting critical information on implementation barriers related to the cultural context within which clinicians work, clinician-specific responsibilities, and the interventions themselves. Since the time this survey was administered, steps have been taken to promote CALM through the implementation of the Zero Suicide program. A number of articles have been written and published on the internal employee website providing education on the value of CALM training accompanied by employee experiences of having taken and implemented the training. At numerous leadership meetings, CALM has been presented as an important educational opportunity for departments and teams across the health system. We have made alterations to the safety plan template in the electronic health record to ensure additional emphasis on lethal means counseling. Additionally, trigger locks for firearms have been made available for distribution in every clinic across our system.

## Limitations

This evaluation had several limitations. First, we did not assess pre- and post-CALM training attitudes and knowledge. Although that was not the purpose of this evaluation, we are limited in our ability to evaluate the impact of CALM training on suicide-prevention-related attitudes and behaviors. Our findings should be interpreted as respondents’ attitudes and behaviors toward suicide prevention and CALM training as collected during the first bi-annual survey of employees following the launch of the Zero Suicide program.

Second, the 2022 Zero Suicide Workforce Survey was voluntary, and compensation was not offered for completing the survey. In this large research-focused academic healthcare organization, leadership approaches survey distribution with a great deal of caution in order to minimize employee survey fatigue. Therefore, system-wide survey requests are voluntary and highly restricted. Although our large sample of employees (3,345) provided ample statistical power for identifying significant differences in the respondents’ attitudes and behaviors toward suicide prevention and CALM training, the rate of included survey responses was low (14.9% of employees). It is possible that the respondents included in our analytic sample and their views may not be representative of the entire population of University of Utah Health employees (i.e., non-response bias). As appropriate, incentives or other initiatives may help increase subsequent survey response rates.

Third, providing education on the importance of CALM in suicide prevention and actually implementing the counseling process with patients are two different components of gaging effectiveness. However, capturing quality assurance data with information not captured in discrete data fields within a medical record can be a complicated and convoluted process. Therefore, we have not yet implemented a quality assurance data extraction process. In March 2023, a discrete field indicating that CALM was done was added to the safety plan built into the University of Utah Health’s electronic health records system. This will allow for easier data extraction in order to track engagement in the CALM process.

Fourth, approximately 56% of survey responses were from direct patient care and 44% reported non-patient care. This is different from the overall employee population within the University of Utah Health system. In May 2022, the University of Utah Hospital and Clinics reported that approximately 80% of employees were involved in direct patient care and 20% of employees were not. Although our results may be skewed towards reflecting clinician attitudes toward CALM training, we stratified our analyzes by those who provide direct patient care. Moreover, our analyzes yield important insights from large samples of both clinician and non-clinician respondents in an academic medical center.

Fifth, it is also important to note that the survey was delivered via employee email, various department specific electronic newsletters, and the system’s home intranet site. Therefore, individuals who completed the survey were more likely to be employees whose roles have them engaged in computer-based activities that allow for time to open and complete a survey. This likely accounts for the higher volume of non-patient care staff and likely leaves staff such as nurses or medical technicians under-represented in this sample. Since social workers, physicians, and other advanced level providers were targeted for CALM training, and nurses and medical technicians were less likely to complete the survey, we are left with a somewhat vague understanding of the suicide prevention knowledge and comfort levels for this employee population who plays a substantive role in direct patient care. Moving forward, efforts will be made to create additional opportunities to elicit more robust employee engagement by involving department and clinic leadership directly. Through continued use of standard electronic methods (i.e., email and newsletter), the use of QR codes in employee break areas, and utilizing department staff meetings, we hope to ensure a more representative sample of employees. Five survey distribution periods will occur every 2 years through 2030.

Sixth, because this workforce survey was conducted 2 years after the launch of the Zero Suicide program, it is possible that respondents included in our analytic sample did not accurately remember certain events or that their attitudes or behaviors may have changed in between the launch of the Zero Suicide program, completing CALM training, and completing the survey (i.e., recall bias).

Finally, our findings reflect the attitudes of the workforce from one academic medical center and may not be generalizable to other healthcare settings located in other geographic locations.

## Conclusion

This evaluation provides important insights into attitudes toward CALM training from the workforce of a large academic medical center implementing a Zero Suicide program. We found that employees who were knowledgeable about suicide warning signs were significantly more likely to have completed CALM training compared to those who were not knowledgeable about suicide warning signs. We also found that employees who were engaged in direct patient care were significantly more likely to complete CALM training compared to respondents not involved in direct patient care. However, the majority of respondents in our analytic sample did not yet complete CALM training, and a non-trivial proportion of respondents did not complete CALM training because they believed CALM was irrelevant to their jobs. This evaluation therefore underscores several important challenges and opportunities for increasing suicide prevention awareness and CALM training in an academic medical center over time, which may ultimately help reduce lethal suicide attempts and save lives.

## Data availability statement

The original contributions presented in the study are included in the article/Supplementary material, further inquiries can be directed to the corresponding author.

## Ethics statement

The studies involving humans were approved by University of Utah Institutional Review Board. The studies were conducted in accordance with the local legislation and institutional requirements. Written informed consent for participation was not required from the participants or the participants’ legal guardians/next of kin because a consent cover letter was provided to participants who completed the survey, which stated that the completion and submission of the survey acted as the individual’s consent to voluntarily participate. This study received an exemption determination from the University of Utah Institutional Review Board for Federal Exemption Categories 2 and 4 defined in 45 CFR 46.101(b) (IRB_00142911).

## Author contributions

RJ: Conceptualization, Investigation, Methodology, Writing – original draft, Data curation, Project administration, Resources. ES: Conceptualization, Data curation, Investigation, Methodology, Project administration, Writing – original draft. EG: Conceptualization, Investigation, Methodology, Writing – original draft, Formal analysis, Software, Visualization.
