# Supplementary material for: Employee attitudes toward suicide prevention and Counseling on Access to Lethal Means: initial findings from an academic medical center implementing the Zero Suicide framework
Source: Front Public Health. 2023 Nov 3;11:1268300. doi: 10.3389/fpubh.2023.1268300 (PMC10654743; doi:10.3389/fpubh.2023.1268300)
Supplement: Supplementary file 1 [file Table_1.DOCX]

Supplementary Material

Employee Attitudes Toward Suicide Prevention and Counseling on Access to Lethal Means: Initial Findings from an Academic Medical Center Implementing the Zero Suicide Framework

Rachael A. Jasperson, LCSW, PhD, MBA^1^, Emily Sullivan, MS, MPH^2^, and Evan V. Goldstein, PhD, MPP^3*^

*** Correspondence:** Evan V. Goldstein, PhD, MPP: evan.goldstein@hsc.utah.edu


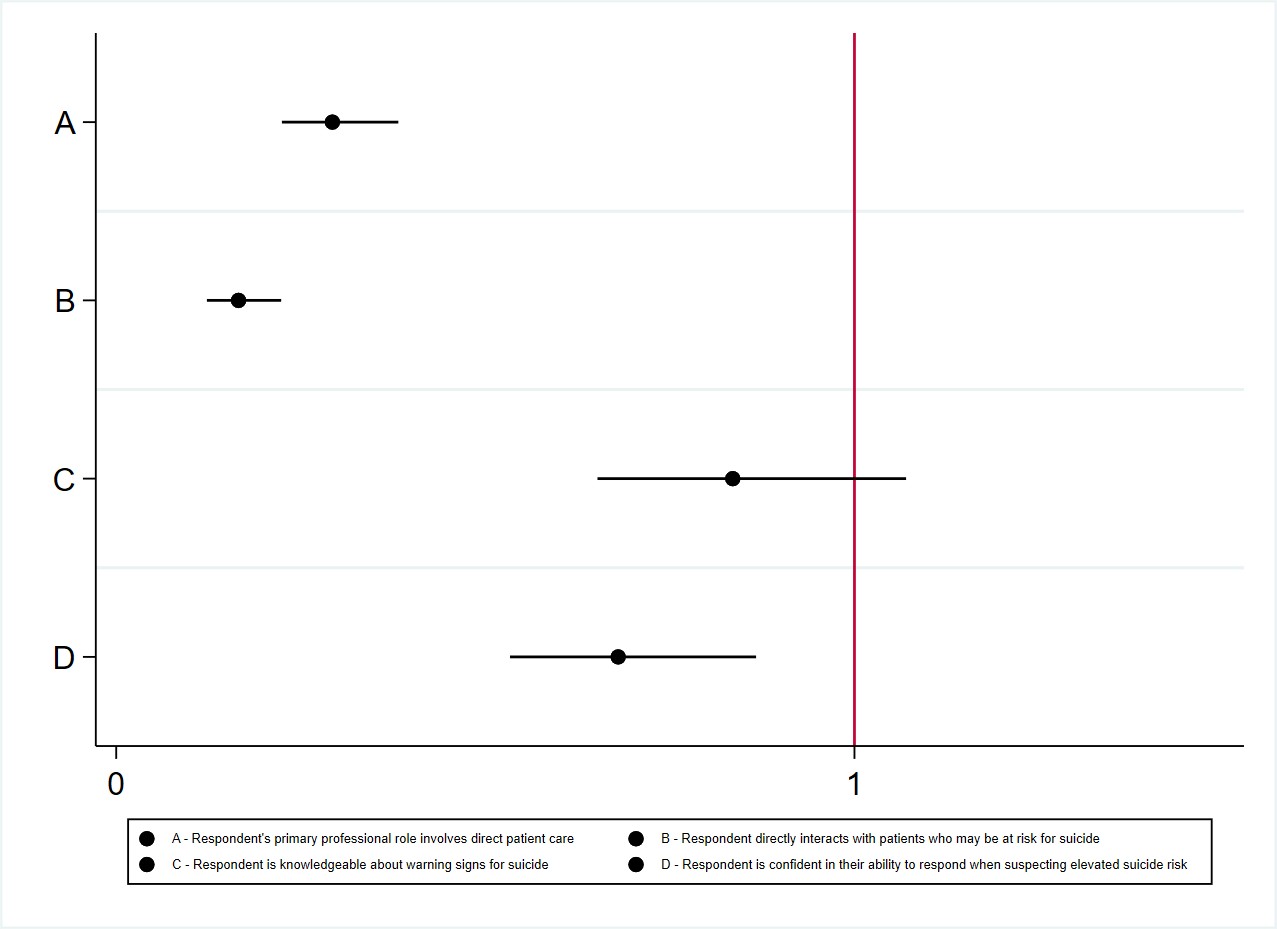


**Supplementary Figure 1. Odds ratios that respondents did not yet complete CALM training because they believe CALM is not relevant to their job by different employee characteristics and knowledge/confidence levels (n = 3,064).**
